# Mutual Links between the Endocannabinoidome and the Gut Microbiome, with Special Reference to Companion Animals: A Nutritional Viewpoint †

**DOI:** 10.3390/ani12030348

**Published:** 2022-01-31

**Authors:** Aniello Schiano Moriello, Vincenzo Di Marzo, Stefania Petrosino

**Affiliations:** 1 Endocannabinoid Research Group, Istituto di Chimica Biomolecolare, Consiglio Nazionale delle Ricerche, Via Campi Flegrei 34, 80078 Napoli, Italy; aniello.schianomoriello@icb.cnr.it (A.S.M.); vdimarzo@icb.cnr.it (V.D.M.); 2 Epitech Group SpA, Via Einaudi 13, 35030 Padova, Italy; 3 Canada Excellence Research Chair on the Microbiome-Endocannabinoidome Axis in Metabolic Health, CRIUCPQ and INAF, Centre NUTRISS, Faculties of Medicine and Agriculture and Food Sciences, Université Laval, Quebéc City, QC G1V 4G5, Canada

**Keywords:** chronic enteropathies, dysbiosis, endocannabinoidome, endocannabinoids, idiopathic inflammation, metabolic disorders, microbiome, neuroinflammation, obesity, oleoylethanolamide, palmitoylethanolamide

## Abstract

**Simple Summary:**

Dysbiosis, which is an imbalance of gut microbial composition and function, can be caused by several external as well as internal factors, contributing to the onset of human and animal disorders, not limited to the gastrointestinal tract. Accordingly, the mechanisms leading to disease development involve a crucial interaction between the gut microbiota, their metabolic products, and the host. The expanded endocannabinoid system, also known as the “endocannabinoidome”, includes endocannabinoids (e.g., anandamide) and endocannabinoid-like mediators (e.g., palmitoylethanolamide), their receptors and metabolic enzymes. Dysregulation of this newly recognized endogenous system is also involved in several diseases. It is becoming increasingly apparent that a link between the endocannabinoidome and the gut microbiome exists. Here, we review some of the latest discoveries related to the functional link between these two complex systems and the disorders emerging from the malfunctioning of such a mutual interaction: for example, idiopathic inflammation, chronic enteropathies, metabolic disease and certain neuroinflammatory disorders. It is expected that in the near future new nutritional tools will emerge based on the expanding knowledge in this cutting-edge field.

**Abstract:**

There is growing evidence that perturbation of the gut microbiome, known as “dysbiosis”, is associated with the pathogenesis of human and veterinary diseases that are not restricted to the gastrointestinal tract. In this regard, recent studies have demonstrated that dysbiosis is linked to the pathogenesis of central neuroinflammatory disorders, supporting the existence of the so-called microbiome-gut-brain axis. The endocannabinoid system is a recently recognized lipid signaling system and termed endocannabinoidome monitoring a variety of body responses. Accumulating evidence demonstrates that a profound link exists between the gut microbiome and the endocannabinoidome, with mutual interactions controlling intestinal homeostasis, energy metabolism and neuroinflammatory responses during physiological conditions. In the present review, we summarize the latest data on the microbiome-endocannabinoidome mutual link in health and disease, focalizing the attention on gut dysbiosis and/or altered endocannabinoidome tone that may distort the bidirectional crosstalk between these two complex systems, thus leading to gastrointestinal and metabolic diseases (e.g., idiopathic inflammation, chronic enteropathies and obesity) as well as neuroinflammatory disorders (e.g., neuropathic pain and depression). We also briefly discuss the novel possible dietary interventions based not only on probiotics and/or prebiotics, but also, and most importantly, on endocannabinoid-like modulators (e.g., palmitoylethanolamide) for intestinal health and beyond.

## 1. The Gut Microbiota and Microbiome

The gastrointestinal (GI) tract of mammals is inhabited by a complex ecosystem of microorganisms, collectively indicated as the gut microbiota, including bacteria, archaea, fungi, viruses and protozoa, whose collective genome (and ensuing proteome and metabolome) is indicated as the gut microbiome [1,2]. In recent years, research involving high-throughput DNA sequencing and shotgun metagenome, proteomics and metabolomics has experienced a true technological revolution and our knowledge about the gut microbiota as well as its role in animal health and disease has grown exponentially [2,3]. Indeed these molecular technologies have allowed the identification of both human and pet unculturable gut bacteria (by far the most abundant microbiota population), currently estimated to range between 10^12^ and 10^14^, outnumbering host cells by several times [4]. Moreover, although feline and canine gut microbiomes remain poorly characterized, the new molecular tools shed some light on phylogenetic and functional similarities between pet and human gut microbiomes [5].

The gut microbiome plays an important role in physiology, metabolism and nutrition of the host, both in humans and animals. In particular, it provides the enzymatic apparatus necessary for the fermentation of non-digestible fibers from plant foods, producing bioactive metabolites, including short-chain fatty acids (SCFAs) such as acetate, propionate and butyrate [6,7,8,9]. The SCFAs play important roles by promoting colonic homeostasis, protecting against colitis [10] as well as stimulating the secretion of glucagon-like peptide-1, thereby overseeing glucose homeostasis [11]. Butyrate is the primary energy source for colonocytes [12] and able to exert anti-inflammatory activity through the inhibition of the nuclear factor NF-κB activation [13], while acetate and propionate are used in the liver for lipogenesis and gluconeogenesis [14]. Finally, butyrate and propionate are also able to promote regulatory T-cell generation in the periphery, in a way dependent, among others, on their histone deacetylase inhibitory activity [15].

**Figure 1:**
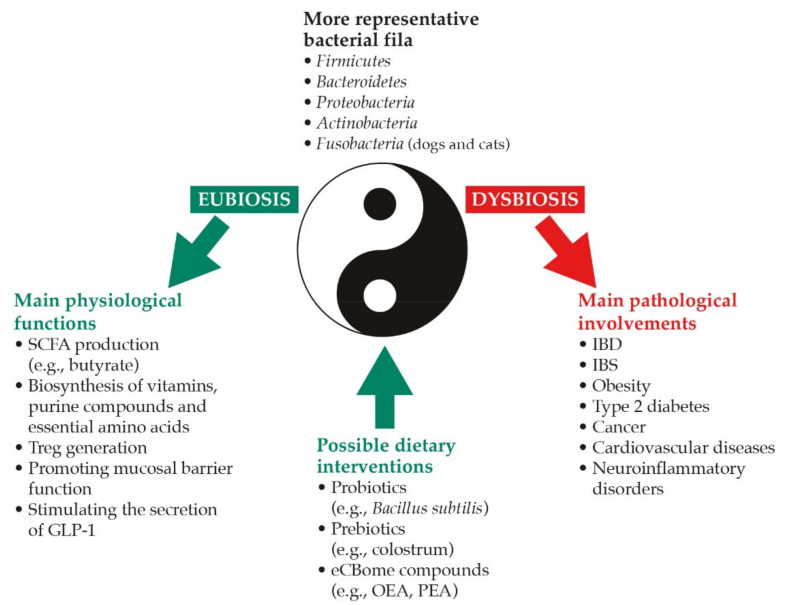
The yin and yang of gut microbiome in intestinal health and beyond. See text for detailed explanation. Abbreviations: eCBome, endocannabinoidome; GLP-1, glucagon-like peptide-1; IBD, inflammatory bowel disease; IBS, irritable bowel disease; OEA, oleoylethanolamide; PEA, palmitoylethanolamide; SCFA, short-chain fatty acid; Treg, regulatory T-cell.

Moreover, the gut microbiome is involved in the biosynthesis of vitamins (such as vitamin K and most of the water-soluble B vitamins), essential amino acids [16,17] and purine compounds that are used for nucleotide biogenesis by the gut mucosa and promote the mucosal barrier function [18] (Figure 1, left side).

It was formerly believed that all mammals are sterile before birth, but recent studies have suggested the presence of microorganisms in the placenta, amniotic fluid and umbilical cord [19,20]. However, it is during birth that newborns are exposed to an extensive number of bacterial microbes through the environment and contact with the mother’s vaginal and fecal microbiomes [21]. In humans, the gut microbial communities change in composition during the first years of life, with variability from baby to baby and depending on infant diet (breast or formula milk) [22]. In the following years (i.e., within the three-year period after birth) the gut microbiome converges in an ‘adult-like’ profile [23,24]. Although studies on the gut microbiome in neonatal dogs and cats are scarce, a similar trend was observed in puppies and kittens, with substantial inter-individual and temporal microbial variability during the early postnatal period [25,26,27]. Similar to humans, early microbial colonization, biodiversity as well as composition of the new-born gut microbiota in puppies and kittens is mainly influenced by vertical transmission from the mother as well as mode of delivery, feeding type and human-pet interaction [7].

In adult humans, the predominant bacterial phyla of the normal gut flora are *Firmicutes*, *Bacteroidetes*, *Proteobacteria* and *Actinobacteria*, with the first two prevailing in healthy adults [28]; whereas the predominant bacterial genera are anaerobic genera, such as *Bacteroides*, *Eubacterium*, *Clostridium*, *Ruminococcus* and *Faecalibacterium* [23]. The core composition of the gut microbiota tends to remain stable in adulthood and change in old age. In fact, in a study carried out on 178 elderly individuals, Claesson and colleagues found a correlation between diet, microbiota composition and health status, indicating a role for diet-driven microbiota alterations in varying rates of health decline upon ageing [29]. The predominant bacterial phyla found in healthy feline and canine gastrointestinal (GI) tract and faecal samples parallel quite well those in humans, with *Firmicutes, Bacteroidetes* and *Proteobacteria* being among the co-dominant phyla [5] (Figure 1, top). *Bacteroides*, *Fusobacterium* and *Prevotella 9* are the dominant genera of canine and feline gut microbiota [30]. The main observed difference is relative to *Fusobacteria*, which usually inhabits—albeit in low proportions—the canine and feline, but not human, healthy gut [5]. Interestingly, a trend toward age-related changes in microbiota is also observed in dogs [31], with lower acetate levels and decreased total SCFAs in faeces compared to adults [32], as well as lower alpha diversity (i.e., species abundance diversity in a given sample) [33]. In dogs, a significant decline in *Fusobacteria* with age was also found by some authors [34]. Of note, age-related gut microbiome composition has been related to short-term memory decline, with better memory performance being associated with a lower proportion of *Actinobacteria* [34]. Finally, although in a different way compared to human beings and dogs, the composition of the feline intestinal microbiota also changes with age [35].

## 2. Dysbiosis and Possible Dietary Interventions

Pathological perturbations of the gut microbial ecosystem balance (the latter known as eubiosis) are defined as dysbiosis [36]. Specifically, dysbiosis can result from the (i) loss of beneficial microorganisms, (ii) excessive growth of potentially harmful organisms, and (iii) reduced overall microbial diversity, with these changes being almost never mutually exclusive [37]. As a result, dysbiosis is able to induce and sustain an inflammatory condition through the predominance of pro-inflammatory microorganisms and the reduction of commensals, promoting immune tolerance mechanisms. The imbalance in the gut microbiota composition has been repeatedly shown to be associated with chronic GI disorders [38,39] and metabolic diseases [40]. In humans, for example, the two main subtypes of human inflammatory bowel disease (IBD), i.e., Crohn’s disease and ulcerative colitis, are associated with dysbiosis characterized by decreased biodiversity and reduced abundance of several types of bacteria belonging to phyla *Firmicutes* and *Bacteroidetes* [41]. This is far from the original hypothesis “one-microbe-one-disease”, while supporting the modern view of an imbalance between the entire gut microbiota and the host as the foundation of several GI (and extra-GI) disorders [37] (Figure 1, right side). 

Although human and pet IBD (the latter better referred to as idiopathic inflammation) differ in certain respects [42], dysbiosis characterizes a variety of GI disorders in pets, too [43,44,45,46,47]. The recently developed fecal canine dysbiosis index has been found to specifically separate healthy dogs from dogs with chronic enteropathies [48]. Interestingly, dysbiosis networks in cats with chronic enteropathies resemble those found in people with IBD [47] more closely than those in dogs [49].

Indeed, dysbiosis has been implicated in a wide range of diseases far beyond IBD. Obesity, diabetes mellitus, cancer, cardiovascular diseases and neuroinflammatory disorders (like, for example, neuropathic pain and depression) are just a few examples of human and pet diseases with clear differences in fecal bacterial composition compared to healthy conditions [37,50,51,52,53,54,55]. Not to mention that aside from the gut microbiome, the skin and oral microbiome are becoming increasingly involved in other human and pet disorders, such as atopic dermatitis [56,57] and periodontal diseases [58,59]. 

Fortunately, the microbiome can be reshaped through different interventions, including nutrition, in order to reverse or attenuate dysbiosis-mediated disorders [37,60]. In this regard, probiotics and prebiotics are widely and successfully used in human and veterinary medicine [61,62,63]. Probiotics are specific live microorganisms that, orally administered in adequate amounts, confer beneficial effects to the host, while prebiotics are selective dietary substrates able to induce changes in the gut microbiota composition and/or activity, with the same aim to benefit the host. Despite the huge variety of different beneficial probiotics, very few are approved for animals. Probiotics, in fact, are feed additives under Regulation (EC) No. 1831/2003 with restrictions on their use. Up to few years ago, only one probiotic strain (i.e., *Enterococcus faecium*) could be used for cats and dogs. Currently, the endospore-forming *Bacillus subtilis* is also admitted and is used in animal feeds, besides being available for humans either as an over-the-counter prophylactic for mild GI disorders or as a health food or nutritional supplement. The advantage of endospore-forming probiotics over probiotics given as vegetative cells is that spore formation provides long-term survival even in extreme environmental conditions. *B. subtilis*, for example, was shown to survive at temperatures ranging from 4 °C to 60 °C and pH from 3 to 11 [64]. *B. subtilis* is a normal constituent of human and canine microbiota [5,9,65], decreases during enteropathies [43,66] and improves intestinal permeability while restoring gut microbiota homeostasis upon supplementation [67,68,69].

On the prebiotic side, studies have shown that dietary supplementation of several different prebiotics exert beneficial effects in IBD through changes in the intestinal microflora composition, i.e., increasing beneficial bacteria such as *Bifidobacteria* and *Lactobacilli* and improving the intestinal epithelial barrier [70]. Bovine colostrum, for example, has repeatedly been shown to (i) inhibit gut pathogens while stimulating the growth of a healthy microbiota (the so-called eubiotic effect) [71,72,73], (ii) reduce iatrogenic injury to the gut [74], (iii) prevent and limit diarrhoea of different origins [74,75]. 

As it will be discussed below, an extensive amount of research is now linking the endocannabinoidome to gut health and microbiome homeostasis was recently reviewed [76]. Dietary interventions with endocannabinoid-like compounds may thus represent a breakthrough in the management of dysbiosis-driven disorders (Figure 1, bottom). 

## 3. The Endocannabinoidome: A Brief Introduction with Special Reference to Energy Metabolism and Gastrointestinal Homeostasis 

The endocannabinoid (eCB) system is a lipid-derived signaling apparatus composed of: (i) G protein-coupled cannabinoid receptors type 1 and type 2, CB1 and CB2; (ii) two main endogenous ligands of such receptors (the so-called endocannabinoids), derived from arachidonic acid, i.e., *N*-arachidonoyl-ethanolamine (AEA or anandamide) and 2-arachidonoyl-glycerol (2-AG), the latter being first isolated from the canine gut [77]; and (iii) the anabolic and catabolic enzymes for the endocannabinoids [78]. AEA and 2-AG are biosynthesized “on demand” through the action of lipases that are stimulated by elevation of intracellular calcium or activation of G proteins [79]. Although the aforementioned molecules are the main and historical components of the eCB system, a great number of other members have also been recognized. In fact, besides AEA and 2-AG, other putative endogenous ligands of CB1 and CB2 receptors have been discovered, such as O-arachidonoyl-ethanolamine (virodhamine), 2-arachidonoyl-glyceryl ether (or noladin ether), oleamide and *N*-arachidonoyl-dopamine [78]. 

*N*-oleoyl-ethanolamine (OEA), *N*-palmitoyl-ethanolamine (PEA), *N*-stearoyl-ethanolamine (SEA) and *N*-linoleoyl-ethanolamine (LEA) are examples of so-called endocannabinoid-like compounds. Although lacking strong affinity for either CB1 or CB2 receptors, they indeed share with AEA a similar chemical structure (i.e., they are all *N*-acylethanolamines, NAEs), as well as the enzymes for the biosynthesis and degradation [78,80,81]. Moreover, together with CB1 and CB2 receptors, other targets have been identified for the endocannabinoids and endocannabinoid-like molecules, such as the transient receptor potential vanilloid type-1 (TRPV1) channel, the G protein-coupled receptor 55 (GPR55) or 119 (GPR119) and peroxisome proliferator activated receptors (PPAR)α and γ [80,81,82]. Consequently, the combination of all these players, including proteins and lipids, led to the expansion of the eCB system into the endocannabinoidome (eCBome), which is currently considered to include about one hundred endocannabinoid-like mediators, more than 20 anabolic and catabolic enzymes and more than 12 receptors [83,84].

With regard to the GI tract, cannabinoid and cannabinoid-related receptors showed wide distribution in several mammals, including mice, pigs, ferrets, dogs, cats, horses and human beings [85,86,87,88,89,90,91,92,93,94,95,96,97,98]. In canine and feline species, CB1 receptor immunolabeling is mainly observed on enteric neurons, nerve fibers, gastric parietal cells, epithelial cells (including goblet cells and enteroendocrine cells) [90,96,97,98]. On the contrary, immunoreactivity for CB2 is generally scanty in epithelial cells, while preferentially observed in perivascular immune cells, e.g., macrophages, B cells and mast cells [90,96,97,98]. GPR55 localizes in smooth muscle cells as well as in lamina propria macrophages, plasma cells, and mast cells [90]. PPARα immunoreactivity is mainly expressed in enteric glia and enteroglial cells [90,96,97], while there is no evidence of PPARγ expression in the canine and feline GI tract (Table 1). It is worth noting that recent studies revealed changes in the expression of some eCBome receptors, i.e., GPR55, CB1 and CB2, during intestinal inflammation and chronic colitis, thus suggesting that eCBome signaling is involved in gut homeostasis [98,99]. Some main aspects are briefly discussed below.

### 3.1. The Endocannabinoidome, Food Intake and Energy Metabolism

The eCBome has a key role in food intake and energy metabolism. The identification of OEA and other NAEs in the GI tract of reptiles and changes in the levels of these lipid compounds during fed compared to fasted conditions confirmed that the eCBome may represent an evolutionarily ancient system in the regulation of energy metabolism [101], as had been suggested by the findings of the role in food intake in invertebrate species (see [102] for review) and in fish [103]. According to several lines of evidence, the eCBome is involved in peripheral glucose and lipid metabolism by controlling the metabolic function of the adipose tissue, liver, endocrine pancreas and GI tract [1]. A dysregulation of the eCB system in these tissues promotes obesity and metabolic syndrome [104,105]. Accordingly, specific correlations between different eCBome players and markers of obesity as well as insulin and glucose homeostasis have been described [106]. For example, food deprivation and re-feeding affect peripheral levels of several eCBome ligands not only in reptiles and fish, but also in mammals, and today the eCBome is confirmed to regulate food intake and energy processing [107]. In human volunteers, plasma levels of NAEs and 2-monoacylglycerols (e.g., 2-AG) correlated with body fat mass and visceral adipose tissue [108]. In particular, NAE plasma levels were found to increase with increased fat mass, whereas circulating 2-AG levels increased with increased visceral fat mass [108]. Self-reported dietary intakes of fatty acids also correlated with plasma levels of 2-AG, omega-3-fatty acid-derived NAEs and 2-monoacylglycerol, irrespective of the body fat distribution [108]. Interestingly, it has been found that a 2-day Mediterranean diet intervention enhances plasma levels of NAEs and 2-monoacylglycerols derived from oleic acid and from omega-3-fatty acids [108]. 

Moreover, in abdominally obese people it was found that plasma levels of 2-AG positively correlated with accumulation of visceral adipose tissue and high triacylglycerol plasma levels [109]. Correspondingly, fasting salivary AEA directly correlated with body mass index, waist circumference and fasting insulin [110] and its plasma levels significantly associated with adiposity [111,112]. On the contrary, body weight loss decreased salivary AEA [110] as well as 2-AG and triacylglycerol plasma levels, visceral adiposity, and insulin resistance [109]. 

Of note, animals with genetic deletion of the NAE biosynthetic enzyme (specifically at the intestinal epithelial level) resulted in obesity and steatosis upon high-fat diet exposure [113]. In these animals, the endogenous levels of NAEs declined—in particular AEA and PEA—and levels of 2-AG were also decreased [113]. Incidentally, the latter result was unexpected (2-AG is not biosynthesized by the deleted pathway) and could have been due to decreased PEA levels [113], given that PEA increased 2-AG in different experimental conditions [114,115]. Insulin resistance, i.e., a hallmark of type 2 diabetes mellitus, was also found to be associated with an imbalanced NAE profile (i.e., reduced PEA/AEA and OEA/AEA ratios) [116]. Moreover, increased levels of AEA (with higher mRNA expression of AEA biosynthetic enzyme and lower expression of the degrading one) as well as decreased 2-AG levels (with increased MAGL expression) were also found in the adipose tissues of diabetic mice, together with increased mRNA expression of CB1 [117,118].

In this scenario, CB1 agonism seems to be the most involved pathway in eCBome regulation of feeding and energy metabolism. In particular, it has been repeatedly reported that CB1 activation increases (i) food intake, adipogenesis and lipogenesis, as well as insulin and leptin resistance in the adipose tissue [105,119,120]; (ii) the expression of enzymes involved in de novo lipogenesis, as well as insulin resistance and dyslipidemia in the liver [121]; and (iii) insulin secretion and trafficking of insulin granules in the endocrine pancreas [105]. On the other hand, CB1 activation reduces gut motility by several mechanisms, including the inhibition of acetylcholine release from cholinergic neurons [122]. 

It is noteworthy that the activation eCBome targets different from CB1 can conversely play beneficial roles during metabolic disorders. In particular, it has been demonstrated that (i) TRPV1 inhibits food intake, improves insulin sensitivity and stimulates thermogenesis [123], (ii) PPARα stimulates fatty acid β-oxidation [124], (iii) GPR55 enhances insulin sensitivity and reduces obesity [125], (iv) CB2 reduces insulin resistance and contributes to the management of diabetes due to its anti-oxidant and anti-inflammatory properties [126], and (v) PPARγ stimulates insulin sensitivity [82]. Accordingly, direct and indirect agonists of these receptors exert modulatory and protective effects on energy metabolism and related disorders. For example, OEA signaling is considered a biosensor for dietary fat [127]. Its production in enterocytes and mobilization in small intestine are stimulated by food intake [128] and in particular, by the release of oleic acid during fat digestion [127]. Newly formed OEA activates PPARα, which leads to satiety possibly through the vagus nerve [127] and the so-called gut-brain axis responsible for controlling food intake [129]. Although less potent than OEA, also LEA was shown to decrease food intake through the same receptor target following oral administration in rodents [130].

Interestingly, two recent clinical trials in obese patients as well as patients with non-alcoholic fatty liver disease showed that OEA supplementation decreased anthropometric measures including body mass index and waist circumference [131,132]. Moreover, dietary supplementation with SEA restores pancreas lipid composition under obesity-induced insulin resistant conditions [133]. Finally, PEA in a bioavailable micronized form has been recently shown to play an important protective role against a hallmark of metabolic disease, i.e., non-alcoholic steatohepatitis [134]. Obesity-related pro-inflammatory states also benefited from PEA administration [135]. The results have been suggested to depend on the anti-inflammatory effects of PEA which have been repeatedly reported not only on immune cells, such as lymphocytes and mast cells [136,137,138], but also specifically on adipocytes [139].

Generally speaking, one may conclude that increased levels of AEA and decreased levels of OEA, PEA and SEA are associated with increased feeding behavior and reduced thermogenesis as well as increased markers of inflammation in adipose tissue and insulin resistance, as recently and extensively reviewed [140,141].

### 3.2. The Endocannabinoidome and Intestinal Permeability Barrier

Accumulating evidence suggests the pivotal role exerted by the eCBome in the pathophysiology of GI disorders [142]. In particular, several ligands and receptors of the eCBome are involved in the regulation of GI motility and secretion, intestinal inflammation and mucosal barrier permeability, as recently reviewed elsewhere [142,143,144]. In this paragraph, description will be limited to the involvement of the eCBome in the so-called “leaky gut”, i.e., the impairment of the gut barrier which is associated not only to different enteropathies (including IBD), but also some of the metabolic disorders we have focused on in the above paragraph, e.g., obesity and diabetes mellitus. 

First, it is interesting to note that mucosal and plasma levels of eCBome mediators change during gut inflammatory conditions. For example, dogs with chronic enteropathies have recently been found to have increased or decreased plasma levels of PEA, 2-AG and AEA, depending on the single compound and the specific enteropathy, i.e., whether it was food-, antibiotic- or immunosuppressive-responsive or belonged to the protein-losing subtype [145]. Moreover, levels of PEA are increased in colon biopsies from patients with coeliac disease and dogs with IBD as well as experimentally induced gut inflammation [100,146,147,148], while markedly decreased in animals fed unbalanced diets [149,150,151]. Interestingly, in mice with genetic deletion of NAE biosynthetic enzyme, a marked inflammatory tone was observed in the basal state, which was believed to result from the observed decline in the levels of PEA [152]. On the contrary, the inhibition of PEA degradative enzyme—which results in the increase of PEA levels in the colon—was found to reduce colon inflammation in two models of IBD [151]. 

Similar findings were observed following PEA oral administration, using either the same [151] or different models of colon inflammation [148,153], with the compound being also able to normalize post-inflammatory increase in intestinal motility [154]. Using labeled dextran it was also shown that oral administration of ultramicronized PEA (i.e., the highly bioavailable and most effective PEA formulation [80,155,156]) significantly counteracted the increased gut permeability in a mouse model of IBD, through either CB2-, GPR55-, or PPARα-mediated mechanisms [153]. 

Moreover, OEA and PEA were effective in preventing the cytokine-induced increased permeability in CaCo-2 cells compared to vehicle, the effect being dependent on TRPV1 and PPARα, respectively [157]. Actually, PEA not only prevented but also reversed the increase in permeability, since it was effective even when applied 72 h after the induction of inflammation [157]. Similar findings were reported with hypoxia-induced permeability in CaCo-2 cells following treatment with OEA and PEA, while treatment with AEA and 2-AG further increased permeability (via CB1 receptors) [158]. In this regard, it is also interesting to note that plasma AEA concentrations in obese subjects were negatively related to duodenal expression of tight junction proteins, suggesting that increased AEA may contribute to altered intestinal permeability in human obesity [111]. 

Summarizing, AEA—like bacterial lipopolysaccharides and inflammatory cytokines—is considered a “gate opener” with regard to gut barrier function, while PEA exerts a beneficial effect on the permeability barrier and is considered a “gate keeper” [140,159].

## 4. The Endocannabinoidome-Gut Microbiome Axis in Intestinal Health and beyond 

**Figure 2:**
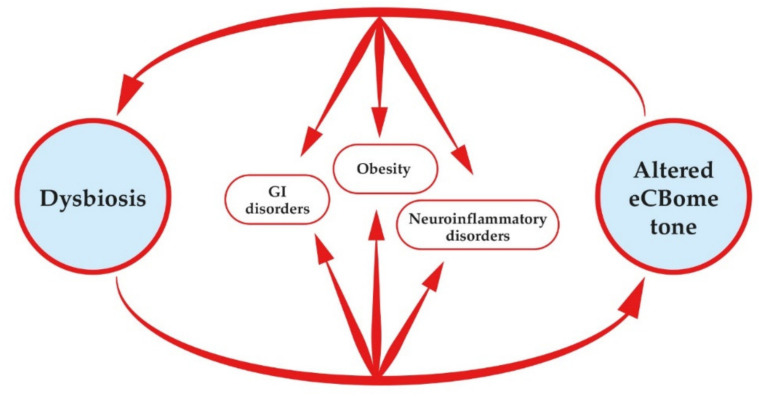
Crosstalk between the gut microbiota and the eCBome. Dysbiosis causes an altered tone of the eCBome, which in turn can feedback on dysbiosis. Disrupted cross talk between these two complex systems is involved in the pathogenesis of gut inflammatory diseases, obesity and neuroinflammatory and mood disorders (e.g., neuropathic pain and depression).

Currently, it is becoming clear that the eCBome and gut microbiota mutually affect each other [1,117,140]. Several lines of evidence are recently suggesting that the altered eCBome tone featuring obesity and diabetes is correlated with gut dysbiosis [127]. Likewise, eCBome is increasingly being considered an important link between the gut microbiome and certain neuroinflammatory disorders [160]. A summary overview is given in Figure 2 and the available evidence is summarized below. 

One of the most interesting studies found that sub-chronic administration of the eCBome mediator OEA changed the faecal microbiota profile of mice fed a normal diet towards a “lean-like phenotype”, shifting the *Firmicutes/Bacteroidetes* ratio in favor of the latter [161]. In different experimental models associated with dysbiosis (e.g., antibiotic treatment, high fat diet-induced obesity), Muccioli and colleagues also highlighted a strict relationship between the colonic eCBome tone and gut dysbiosis [162]. In particular, perturbations of gut microbiota or genetic disruptions of the gut barrier reduced colonic mRNA expression for CB1 and changed the mRNA expression of eCB hydrolases (i.e., eCB degrading enzymes), with the main result being the increase of AEA levels [162]. Prebiotic supplementation reduced gut permeability in high-fat-diet-induced obese mice, a similar finding being observed when a selective antagonist of CB1 was used [162]. Prebiotic administration to obese mice also increased mRNA expression of AEA hydrolase in the adipose tissue, correspondingly decreasing local levels of AEA [162]. Interestingly, these changes were associated with reduced adipocyte differentiation and lipogenesis, as well as less fat mass development in obese mice [162]. Again, similar findings were observed when a selective CB1 antagonist was used [162]. In line with these findings, decreased mRNA expression of CB1 and CB2 in enterocolic biopsies from privately-owned dogs with colonic dysmotility disturbances was significantly counteracted by a 90 day-probiotic treatment [98].

Furthermore, some interesting data come from studies on bacteria genetically engineered to biosynthesize the precursor for NAE biosynthesis. Once incorporated into the gut microbiota they exerted a protective function against obesity, provided that a sufficient active anabolic enzyme was present in either the host or engineered bacteria [163,164].

In addition, a bidirectional relationship between eCBome tone and gut dysbiosis has been suggested in atherosclerosis [162,165] and diabetes [117,118]. In particular, in the adipose tissues of diabetic mice, specific changes in the composition of the gut microbiota were observed concurrently with changes in the eCBome tone (mainly increased AEA and decreased 2-AG levels) [117,118]. 

Some interesting evidence on the role played by NAEs on gut microbiota also comes from studies on specific genetic deletion of eCB anabolic pathways. In particular, under a control diet, animals with an adipocyte-specific deletion of the NAE biosynthetic enzyme developed a shift in the composition of the gut microbiota together with an obese phenotype (i.e., deranged adipose and whole-body lipid metabolism, altered browning process, insulin resistance and glucose intolerance) [152]. Long-term antibiotic treatment significantly reversed all the alterations, whereas transferring gut microbiota to germ-free mice partially replicated them [152]. Similar results were observed in animals with genetic intestinal deletion of the NAE biosynthetic enzyme [113]. Upon high-fat diet exposure, these animals not only developed obesity and steatosis, but dysbiosis was also observed [113]. When a protective bacterium, i.e., *A. muciniphila* [166,167], was administered, it maintained its efficacy against obesity and metabolic syndrome, suggesting that intestinal NAEs did not affect the probiotic efficacy in this condition [113]. 

The role of 2-AG on the gut microbiota has also been recently investigated. Mice with monoglyceride lipase deletion not only presented with higher intestinal levels of 2-AG and congeners [168], but were also resistant to diet-induced obesity and metabolic disturbances, and most importantly they exhibited changes in gut microbiota [169]. Conversely, ablation of gut microbiome in germ-free mice resulted in intestinal eCBome changes at either the level of receptors (e.g., CB1, PPARα and GPR55), enzymes or NAEs, regardless of age and gender [170]. These changes could be entirely of partially reversed following fecal microbiome transfer from conventionally raised mice [170]. 

Finally, a recent ex vivo study has reported that bacterial monocultures treated with a specific and highly concentrated NAE cocktail including AEA, LEA, OEA and PEA, promoted the growth of microbial species found to be over-presented in IBD, while reducing the growth of those depleted in IBD [171]. The study thus suggested that NAEs strongly affect bacterial growth and reflect altered bacterial abundances associated with IBD pathogenesis [171]. Although very interesting, these findings are apparently inconsistent with the protective effects played by NAEs in gut disorders (see above); the difference could partly depend on the fact that in vivo complexity was not sufficiently recapitulated by the ex vivo setting. Even more so if one considers that a remarkable switch in the NAE precursors was found after administration of engineered NAE-producing bacteria as a function of the type of dietary fatty acids [172]. The results of this study also appear to be in contrast with a recent report, where, using data from a 6-week exercise intervention and a cross sectional validation cohort of obese/overweight individuals, baseline serum levels of AEA and OEA were positively associated with alpha diversity as well as SCFA producing bacteria such as *Bifidobacterium*, *Coprococcus 3* and *Faecalibacterium*. Additionally, AEA was positively associated with butyrate. Serum AEA, OEA and PEA all increased significantly with exercise and changes in AEA correlated with butyrate, whereas increases in AEA and PEA correlated with decreases in TNF-α and IL-6. It was calculated that these two NAEs mediated one third of the effect of SCFAs on these cytokines [173]. Whilst very interesting, because carried out in obese/overweight volunteers, this study seems to contrast with the concept, discussed above, that while PEA counteracts inflammation, AEA may worsen it. Clearly, the overall pro-inflammatory or anti-inflammatory action of AEA depends on the receptors it modulates (respectively, CB1 and GPR55, on the one hand, or TRPV1 and CB2 on the other hand), which in turn might depend on the baseline context under study (such as exercise or the presence or lack of obesity/overweight).

Taken together, the findings discussed above suggest the eCBome-gut microbiome axis plays a key role in intestinal and metabolic health [169], with gut microbiome controlling the eCBome tone and vice versa [170].

### 4.1. Diet, Microbiome and Endocannabinoidome Tone 

Currently, only few studies have investigated the possible link(s) between diet-induced perturbations of gut microbiota profile and changes in the eCBome tone. Lacroix and colleagues demonstrated that high fat-high sucrose diet not only lead to glucose intolerance, obesity and hyperinsulinemia in mice, but also altered the gut microbiota profile as well as the intestinal and serum eCBome tone [174]. In particular, they found that during high fat-high sucrose diet low abundance of metabolically beneficial genera correlated with increased ileal levels of AEA and plasma levels of both AEA and 2-AG [174]. Accordingly, ileal mRNA expression of AEA and 2-AG degrading enzymes was decreased, whereas the expression of 2-AG biosynthesizing enzyme was increased [174]. Moreover, decreased mRNA expression for either PPARα or CB2 was found [174]. Correlation analyses suggested that interactions between gut microbiome and eCBome not only exist but could also affect the development of dysbiosis as well as diet-induced metabolic disturbances [174]. 

In addition, Castonguay-Paradis and colleagues have recently demonstrated that the abundance of some gut microbiota taxa in human subjects is associated with increased plasma levels of NAEs and 2-monoacylglycerols, which, and particularly those derived from omega-3 fatty acids, in turn correlated positively with the dietary intake of the respective fatty acids, irrespective of fat mass [108]. According to the authors, the finding suggests that dietary interventions aimed at properly manipulating the eCBome tone may counteract metabolic disturbances linked to gut dysbiosis [108].

### 4.2. Microbiome-Gut-Brain Axis and Endocannabinoidome: “Omics” Interactions go Central 

Accumulating evidence is suggestive of the eCBome linking the gut microbiome to central nervous system pathophysiology. One excellent example is pain perception in vitamin D deficient mice [175]. It has recently been found that these animals concurrently present marked dysbiosis, with lower microbial diversity, together with tactile allodynia and neuronal hyperexcitability [175]. Most notably, vitamin D deficient mice also showed changes in the eCBome at both spinal and colon level (e.g., increased AEA levels) [175]. Interestingly, treatment with ultramicronized PEA reversed chronic pain and neuronal excitability normalized spinal eCB changes and increased some specific commensal gut bacteria, in particular *A. muciniphila* [175], known to exert intestinal protective effects [113,166,167]. More importantly, the results suggested that, at least in part, the analgesic effects of ultramicronized PEA were peripheral in nature and dependent on gut microbiota [175]. 

A further example of the involvement of gut microbiome-eCBome interactions in central neuroinflammatory disorders is depression. Among other psychiatric disorders, depression is indeed associated with dysbiosis, intestinal inflammation and loss of gut integrity [176,177]. In particular, in a mouse model of antimicrobial cocktail-induced dysbiosis, depressive behaviors and reduced social recognition memory as well as increased of gut inflammation were observed [178]. Notably, these changes were also accompanied by increased biochemical and functional changes at hippocampus level, including activation of astrocytes and microglia; moreover, hippocampal and gut alteration of some eCBome members were evident [178]. In fact, increased TRPV1 phophosphorylation/sensitization was observed in the hippocampus, whereas decreased levels of *N*-acyl-serotonins (i.e., TRPV1 antagonists and inhibitors of NAE degradation) were found in the small intestine [178]. Importantly, probiotic supplementation counteracted the depressive-like behavior, normalized social activity and reduced gut inflammation as well as biochemical and functional hippocampal alterations, while reverting the decrease of gut *N*-acyl-serotonin levels [178]. 

From a large cohort study on nearly 800 volunteer twins it has recently emerged that the eCBome mediates the relationship between gut microbiome and anhedonia/amotivation [179]. In particular, the authors tested the hypothesis that either reduced serum levels of PEA or increased stool levels of PEA would mediate the association between microbial diversity and anhedonia/amotivation. Indeed, the association was found to be mediated by faecal, but not serum, levels of PEA [179].

Additionally, many studies have reported gut dysbiosis to be associated with autism spectrum disorders [180,181]. Again, eCBome-microbiome interactions seem to play a role, as recently suggested by a study in BTBR mice, a strain displaying autistic-like features, including social deficits and repetitive behavior [182]. Administration of ultramicronized PEA improved the altered behavioral phenotype, the effect being dependent on PPARα activation [182]. Ultramicronized PEA also restored hippocampal mitochondrial function and decreased the expression of pro-inflammatory cytokines at hippocampal, serum, and colonic level [182]. Importantly, gut permeability and faecal microbiota profile showed improvements following PEA administration, with the main finding being the rise of *Firmicutes/Bacteroidetes* ratio, mainly due to the increase of butyrate-producing *Clostridiales* [182]. Taken together, the results suggested that PEA (i) controlled neuroinflammation, (ii) exerted anti-inflammatory effects at colonic and systemic level, (iii) restored gut homeostasis by improving gut integrity and remodeling gut microbiota composition [182]. 

It is finally worth mentioning the mutual nature of eCBome-microbiome interactions in central nervous system (CNS) pathophysiology, i.e., not only that the eCBome controls microbiome, but that the reverse also holds true. Manca and collaborators have indeed recently shown that the overall impaired eCBome signaling observed in the brain of germ-free mice was attenuated by fecal microbiome transfer from conventionally raised mice [183]. 

The eCBome tone may thus play unexpected roles not only in gut homeostasis and energy metabolism, but also in the CNS consequences of dysbiosis.

### 4.3. Microbiota as a Potential Source of Endocannabinoidome Mediators

An expanding theme is the potential capability of commensal microorganisms to affect eCBome signaling by directly producing NAE-like molecules able to bind the host G-protein–coupled receptors. *N*-acyl-3-hydroxypalmitoyl-glycine, called Commendamide, is the first of these molecules to be identified [184,185]. *N*-oleoyl serinol is a further member of microbiota-encoded NAE family. In particular, it is produced by commensal bacteria and acts as GPR119 agonist, sharing a similar structure as well as mechanism with OEA which actually activates GPR119 [186]. *N*-oleoyl serinol has been found to regulate metabolic hormones and glucose homeostasis as efficiently as OEA [186]. Additionally, it has been discovered that some microbiota-derived molecules may also act through the host TRPV1 (the 2021 Nobel Prize-winning receptor). In particular, a linoleic acid metabolite produced by gut lactic acid bacteria, i.e., 10-oxo-12(Z)-octadecenoic acid also referred to as KetoA, was able to augment energy metabolism through the activation of TRPV1 channels, thus protecting mice from diet-induced obesity and ameliorating obesity-associated metabolic disorders [187]. Finally, gut *Clostridia* were very recently shown to conjugate some neurotransmitters or neurotransmitter-like molecules, such as dopamine, tyramine and tryptamine, with diet- and human-derived fatty acids to produce long chain fatty acid amides that modulate the activities of host GPCRs, including some eCBome receptors [188].

## 5. Conclusions

In summary, the data reviewed in this article clearly point to the existence of an eCBome-gut microbiome axis. The malfunctioning of this axis may be involved in a variety of disorders wherein intestinal dysfunction plays a role, such as obesity, chronic inflammatory enteropathies as well as neuroinflammatory disorders. Mediators of the eCBome and their receptors appear to influence the complex and still largely unexplored communication between the host and its gut microbiome. The design and development of eCBome receptor agonists, antagonists and allosteric modulators as well as anabolic/catabolic enzyme inhibitors may thus represent future therapeutic interventions for gut dysbiosis-driven diseases. Likewise, targeting gut microbiome with dietary interventions (e.g., prebiotics, probiotics) may be of potential use for the prevention and treatment of disorders related to eCBome dysfunction. In this scenario, food for special medical purposes and dietetic complementary feeds (for human and veterinary use, respectively) containing ultramicronized PEA—alone or in combination with probiotics and prebiotics—may be readily available nutritional tools to keep pathological alterations of the eCBome-gut microbiome axis under control. It is expected that in the near future new nutritional tools will emerge based on the expanding knowledge in this cutting-edge field. In particular, balanced diets containing amounts of fatty acid precursors for eCBome mediators with different biological activities (such as PEA and AEA, for example), could be designed, based on the increasingly accepted concept that dietary fatty acids are a strong determinant of plasma and tissue levels of such mediators [108,189]. These options will hopefully represent valuable and safer alternatives to current treatments, such as antibiotics for chronic enteropathies, which not only cause long-term negative alterations of gut microbiota but are also a global concern (i.e., because of the development of antibiotic resistance), both in human and veterinary medicine [190,191,192].

## Figures and Tables

**Table 1:** Main distribution of the investigated eCBome receptors in the gastrointestinal (GI) tract of either dogs (grey), cats (yellow) or both species (green). Modified from [98,100].

Cell Type	CB1	CB2	GPR55	PPARα	PPARγ
Lamina propria cells					
Enterocytes/Colonocytes					
Mast cells					
Immunocytes					
Smooth muscle cells					
Macrophages					
Goblet cells					
Submucosal plexus neurons and glia					
Myenteric plexus glia					
Myenteric plexus neurons					
Enteroendocrine cells					
Enteric neurons					
Enteroglial cells					

## Data Availability

Not applicable.
